# Nocardiosis: Risk Factors, Clinical Characteristics and Outcome

**DOI:** 10.5812/ircmj.2384

**Published:** 2013-05-05

**Authors:** Ilad Alavi Darazam, Masoud Shamaei, Mandana Mobarhan, Shahin Ghasemi, Payam Tabarsi, Masoud Motavasseli, Davood Mansouri

**Affiliations:** 1 National Research Institute of Tuberculosis and Lung Diseases, Shahid Beheshti University of Medical Sciences, Tehran, IR Iran; 2 Internal Medicine Department, Tehran University of Medical Sciences, Tehran, IR Iran

**Keywords:** Nocardia Infections, Immunocompromised Host, Treatment Outcome, Nocardiosis, Cutaneous

## Abstract

Nocardiosis has been reported increasingly in recent two decades, probably due to improvement in isolation of the organism and increased burden of immune compromised patients. Nocardia occasionally has been reported in healthy people. A case series of definitive Nocardiosis (2002 to 2010), clinical characteristics, underlying diseases, immune status and in-patient outcome were studied in a tertiary referral center. Twenty one patients with definite diagnosis of Nocardiosis were studied. 17 cases (81%) had an underlying disease (diabetes mellitus, corticosteroid therapy, and chronic granulomatous disease and collagen vascular diseases). Four patients (19%) were immune-competent without any predisposing disease. In 17 patients (81%), Nocardiosis was limited to respiratory tract and in 4 cases (19%) it was disseminated with multi organ involvement. Two cases (9.5%) died in hospital.

## 1. Background

Nocardiosis remains as a possible cause of pulmonary and systemic infection in ‎immunocompromised patients but it can be isolated in otherwise immune-competent patients. ‎Systemic involvement can be seen in immune-competent patients. ‎Nocardiosis is an uncommon gram-positive bacterial infection in human and animal caused by aerobic actinomycetes in the genus Nocardia (1). There is limited data about true worldwide incidence of Nocardia infection. Annual incidence of Nocardiosis in United States is about 500-1000 cases per year (2). Difficulties in isolation of the organism and incubation experience and lack of systematic report for Nocardia infection in health system is probable reasons (2).

Isolated pulmonary, central nervous system or cutaneous involvement and disseminated or systemic infection are major clinical classification of Nocardiosis (1, 3-5). Nocardia infection usually occurs in patients with impaired immunity. Phagocytic and cell mediated immunity have important role in protecting against Nocardia infection particularly disseminated disease (6). There is no evidence that B cells directly influence host defenses in Nocardia infection (7). Nocardia infection is known as an opportunistic infection in immunocompromised hosts but it consisted at least 15% of the infections in patients without a definable predisposing condition (2). HIV infection, alcohol use, organ transplantation, lymphoreticular neoplasms are the most frequent conditions can leading to disseminated Nocardiosis (6, 8). Advanced chronic obstructive lung diseases and autoimmune diseases are other risk factors for Nocardia infections (9, 10). Establishing a diagnosis of Nocardiosis is problematic since an invasive procedure is often required to obtain an adequate specimen and recovery of Nocardia in the laboratory is difficult because of its fastidious nature and slow growth, so it is very important to inform the microbiology laboratories when Nocardiosis is suspected (5).

## 2. Case Report

All definite Nocardiosis patients and their clinical characteristics, probable underlying diseases and predisposing factors, complications and also early outcome of patients in hospital including complications, clinical response and mortality were reviewed in 8 year duration compared to Nocardiosis in otherwise healthy patients without underlying factors. In a case series study, the patients with Nocardia infection were reviewed according to isolation of microorganism from culture of suspected sites including bronchoalveolar lavage (BAL), computed tomography guided biopsy of lung, open lung biopsy, skin ulcer biopsies and the other possible involved organs. Patients were enrolled in the study from 2002 to 2010 and possible cases without definite diagnosis of Nocardiosis were excluded.

Aerobic incubation, culture of tissue sample obtained from the patients showed rough, yellowish colonies. Modified acid-fast bacilli stain demonstrated filamentous, partially acid-fast, gram-positive bacteria with the characteristic branching and beaded appearance of Nocardia species (the isolates were further characterized as Nocardia asteroides). Patients were classified according to their characteristics including age, gender, underlying diseases and predisposing factors, clinical manifestations, involved organs and outcome. Outcome of patients was evaluated according to response to treatment (clinical resolution of pulmonary and central nervous system manifestations with or without imaging study and healing skin ulcers), complications and mortality rate in hospital. Definite Nocardiosis was established in twenty one patients who were referred to this center. Seventeen of them were male and mean age of patients was 37.9 ± 19.66 with range of 9 to 74.

Predisposing factors or underlying diseases were diagnosed in seventeen cases (81%). Three patients had previously diagnosed chronic granulomatous disease and Nocardiosis was limited to lung. Four patients had collagen vascular (CVD) and autoimmune disease including Systemic Lupus Erythematosus (SLE) (2 cases), one Behcet's disease and polymyositis. Among the patients with CVD, three had been previously diagnosed and were taking steroids when nocardiosis occurred and the other one presented with new onset diffuse alveolar hemorrhage and was diagnosed as lupus capillaritis and simultaneous nocardia infection. Nocardia associated pulmonary empyema was prominent manifestation of two patients (SLE and Behcet's disease).

One of the patients was a 50 year-old man who used 100 mg Prednisolone since two months before admission for weight gain. He presented with disseminated multiple cavitary pulmonary lesions and central nervous system involvement. Uncontrolled diabetes mellitus type 2 was an underlying disorder in 3 cases; one of them had associated human immune deficiency virus (HIV), hepatitis C (HCV) and tuberculosis infections. History of tuberculosis and pulmonary parenchymal scars was seen in one patient with disseminated nocardiosis including multiple skin ulcers, pulmonary lesions and multiple brain abscesses. She expired due to CNS involvement despite neurosurgical drainage.

Lymphoma, Cytomegalovirus infection and Aspergillosis were seen in another case with multiple pulmonary cavities and low CD4 count diagnosed as idiopathic CD4 T lymphocytopenia. Cavitary pulmonary lesions due to Nocardiosis were seen in a 12 year-old child with underlying pulmonary alveolar proteinosis (PAP). Other respiratory diseases including bronchiectasis and chronic obstructive pulmonary disease were risk factors for Nocardiosis in 3 patients. Overall 17 patients had isolated respiratory tract Nocardiosis and 4 cases presented with disseminated Nocardiosis including lung disease with CNS infection and or cutaneous ulcers.

Early diagnosis and treatment with proper drug regimen in 19 patients (90.5%) resulted in complete resolution of infection. Only two cases (9.5%) died, one of them due to multiple brain abscess and CNS complications and the other due to progressive muscular weakness secondary to underlying polymyositis and respiratory failure. As mentioned above, four patients (19%) had not any underlying factor despite complete immunologic investigations. Among four immune-competent patients one had pulmonary and cutaneous disease and the other had multiple organ involvement consisted of lung, brain, skin and sacroiliac joint infection, two patients had isolated pulmonary disease.

## 3. Discussion

Immunocompromised patients were the most cases of nocardia infections observed in this series. Most of them had pulmonary involvement and it was predictable due to prevalence of pulmonary disease in Nocardiosis documented in several studies and also this center is a tertiary pulmonary diseases hospital. Report of three cases with chronic granulomatous disease can also be considerable. Other finding was occurrence of disseminated disease in two cases of four immune -competent reported in this study. In a comparative study of organ transplant patients with 70 matched control subjects, high-dose steroids, cytomegalovirus disease and a high median calcineurin inhibitor level were found to be independent risk factors for Nocardia infection (11). A review of Nocardiosis patients in a large hospital in South Florida over 6 years has described 25 cases that 76% of them were infected with HIV (12). In another observational study of all the patients diagnosed with pulmonary Nocardiosis over a 13-year period the predisposing conditions were COPD (23%) (13, 14), transplantation (29%), HIV infection (19%), alcoholism (6.5%) and treatment with steroids (64.5%). Dissemination to the central nervous system was also related to alcoholism (13).

Patients with PAP have an increased risk of superinfection with opportunistic organisms such as Nocardia and Mycobacterium due to impaired macrophage and neutrophil function (15). In other hand acquired PAP can be seen with some underlying causes like Nocardia and Mycobacterium tuberculosis infections (16). Other predisposing diseases like primary immune deficiencies are known to detect with Nocardia as opportunistic infection (17, 18). Chronic granulomatous disease was underlying disease in three cases of this study. Direct inoculation of the organism as a result of trauma, surgery, vascular catheters, and animal scratches or bites is most probable cause of isolated cutaneous disease (19). Nocardiosis can occur in apparently healthy population but further detailed immunologic evaluation particularly considering interleukin12-gamma interferon pathway deficiency or other immunologic systems may help in diagnosis of these patients’ underlying diseases in the future. Occasionally isolation of Nocardia is due to colonization, but clinical decision making to receive treatment is according to apparent Nocardia related disease and existence of immune deficiency and comorbidity. However, pulmonary colonization in healthy people without disease is not rare (4, 20).

Drug of choice for Nocardia is Trimethoprim-sulfamethoxazole (TMP-SMX), but other regimens like Amikacin, Imipenem, Minocycline, Linezolid and Cephalosporins are alternatives (4, 21-23). Occurrence of Nocardia in patients on TMP-SMX prophylaxis may happen rarely, which means that this drug is not fully protective agent against Nocardiosis (10, 21). Almost all of patients in this case series were treated with TMP-SMX and a few cases who were in other regimens after diagnosis of Nocardiosis changed to and continued by TMP-SMX. According to the site and extent of infection and predisposing factors clinical outcome for Nocardiosis is different. Frequently patients with soft tissue infection have a complete cure compared with 90 % cure in pleuropulmonary disease, 63% in disseminated infection and 50% in brain abscess particularly in immunocompromised patients (24, 25). It seems that mortality is higher in patients receiving corticosteroid or antineoplastic agents but is not related to the severity of the underlying disease (26).

In this series one patient with brain abscess and the other one with pulmonary disease due to advanced underlying polymyositis died despite of appropriate medical treatment. Other patients were treated and cured completely. Outcome and mortality findings were similar to previous studies. In conclusion, Nocardia infection must be considered in patients with complicated pulmonary involvement and also simultaneous cutaneopulmonary lesions, including healthy people without marked immune deficiency. It is obvious that in patients with underlying diseases Nocardia remains a cause of complicated pulmonary and disseminated infection (Table 1).

**Table 1.** Patients’Characteristics, Underlying Diseases, Clinical Response and Survival in Hospital, and Organ Involvement (Pulmonary or More Than one Organ Involvement)

	Age	Gender	Underlyig Disease	Type of Underlying Disease	Outcome	Organ Involvement
**1**	18	male	yes	CGD ^a^	survived	pulmonary
**2**	23	male	yes	Broncheictasis	survived	pulmonary
**3**	41	male	yes	Behcet, CS ^a^	survived	pulmonary
**4**	48	male	yes	CS	survived	systemic
**5**	63	male	yes	Uncontrolled DM ^a^	survived	pulmonary
**6**	17	female	yes	SLE ^a^	survived	pulmonary
**7**	60	female	yes	Polymyositis	expired	systemic
**8**	51	female	yes	Healed Tuberculosis	expired	pulmonary
**9**	38	male	yes	DM, TB, HIV positive, HCV positive	survived	pulmonary
**10**	23	male	yes	CGD	survived	pulmonary
**11**	20	male	yes	Idiopathic CD4 T lymphocytopenia	survived	pulmonary
**12**	62	male	yes	COPD ^a^	survived	pulmonary
**13**	57	male	yes	Uncontrolled DM	survived	pulmonary
**14**	32	male	yes	SLE	survived	pulmonary
**15**	58	male	yes	Bronchiectasis	survived	pulmonary
**16**	25	male	no	-	survived	pulmonary
**17**	74	male	no	-	survived	systemic
**18**	9	male	no	-	survived	systemic
**19**	45	female	no	-	survived	pulmonary
**20**	12	male	yes	PAP ^a^	survived	pulmonary
**21**	20	male	yes	CGD	survived	pulmonary

^a^Abbreviations: CGD: Chronic granulomatous disease; COPD, Chronic obstructive pulmonary diseases; CS, Corticosteroid; DM, Diabetes mellitus; PAP, Pulmonary alveolar proteinosis; SLE, Systemic lupus erythematous
